# A Review of CRISPR Tools for Treating Usher Syndrome: Applicability, Safety, Efficiency, and In Vivo Delivery

**DOI:** 10.3390/ijms24087603

**Published:** 2023-04-20

**Authors:** Lauren Major, Michelle E. McClements, Robert E. MacLaren

**Affiliations:** 1 Laboratory of Ophthalmology, Nuffield Department of Clinical Neurosciences & NIHR Oxford Biomedical Research Centre, University of Oxford, Oxford OX3 9DU, UK; 2 Oxford Eye Hospital, Oxford University Hospitals NHS Foundation Trust, Oxford OX3 9DU, UK

**Keywords:** CRISPR/Cas9, base-editing, prime-editing, Usher syndrome, genome engineering, gene therapy

## Abstract

This review considers research into the treatment of Usher syndrome, a deaf-blindness syndrome inherited in an autosomal recessive manner. Usher syndrome mutations are markedly heterogeneous, involving many different genes, and research grants are limited due to minimal patient populations. Furthermore, gene augmentation therapies are impossible in all but three Usher syndromes as the cDNA sequence exceeds the 4.7 kb AAV packaging limit. It is, therefore, vital to focus research efforts on alternative tools with the broadest applicability. The CRISPR field took off in recent years following the discovery of the DNA editing activity of Cas9 in 2012. New generations of CRISPR tools have succeeded the original CRISPR/Cas9 model to enable more sophisticated genomic amendments such as epigenetic modification and precise sequence alterations. This review will evaluate the most popular CRISPR tools to date: CRISPR/Cas9, base editing, and prime editing. It will consider these tools in terms of applicability (in relation to the ten most prevalent *USH2A* mutations), safety, efficiency, and in vivo delivery potential with the intention of guiding future research investment.

## 1. Introduction

Research into rare diseases often encounters barriers to funding due to the lack of commercial interest in small patient populations. Consequently, it is important to develop tools that have broad applicability. Usher syndrome is a rare disease with a prevalence that varies according to locality—in the USA, estimated at ~4.4:100,000 [1]. This review focuses on Usher syndrome due to its frequency among inherited retinal degenerations and the lack of current treatment modalities. Moreover, the eye is an appealing target due to its accessibility, relative immune privilege, and numerous clinical outcome measures.

**Figure 1:**
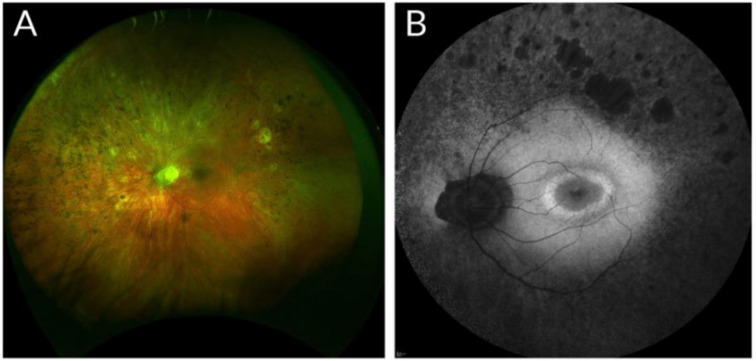
Fundus images of a patient with compound c.2299delG/c.2276G>T *USH2A* mutations. (**A**) Colour fundus photo demonstrating peripheral pigmentary retinopathy, retinal thinning, and vascular attenuation. (**B**) Fundus autofluorescence image demonstrating peripheral hypo-autofluorescence and patches of RPE atrophy. A hyper-autofluorescent ring at the macula is present. Images from Oxford Eye Hospital, Oxford University Hospitals NHS Foundation Trust, Oxford.

Usher Syndrome, first described by Charles Usher in 1914 [2], is a deaf-blindness syndrome inherited in an autosomal recessive manner. Patients are placed into one of three categories based on the severity of their signs. Type 1 Usher patients display the most debilitating signs with vestibular dysfunction, pre-pubertal onset of retinitis pigmentosa (RP), and pronounced congenital hearing deficits. Usher Syndrome type 2 patients typically have preserved vestibular function, moderate congenital hearing loss, and RP onset before the third decade of life (Figure 1). Type 3 Usher patients have greater variability with respect to vestibular function and severity/onset of RP. They display congenital or early onset progressive hearing impairment and are concentrated mainly in Finland [3,4].

The focus of the review is Usher Syndrome Type 2A since mutations within *USH2A* account for over 50% of Usher Syndrome cases and up to 79% of Usher 2 patients [5,6]. Three genes have been associated with Usher Syndrome type 2: *USH2A*, *ADGRV1,* and *WHRN*, which together comprise the periciliary membrane complex at the junction between the inner and outer photoreceptor segment [1]. The mutations within *USH2A* result in a non-functional protein. The function of usherin, encoded by the *USH2A* gene, is not fully understood. However, some purported ideas include a role in transducing mechanical stress, docking/membrane fusion of post-Golgi vesicles, structural maintenance, or acting as a diffusion barrier between the cell body and connecting cilium [7,8]. Regardless of the exact mechanism, pathogenic mutations within *USH2A* result in the defective development of cochlear hair cells and the maintenance of photoreceptor cells [1].

Some gene therapeutics targeting Usher syndrome have progressed to clinical trials. The gene replacement therapy ‘UshStat’ delivers *MYO7A* cDNA within a lentiviral vector into Usher type 1 patients. The trial reached phase 1/2a (NCT 01505062), however, was prematurely terminated. The ProQR trial has made more headway: the antisense oligonucleotide (QR-421a) binds to an mRNA splice site, causing the translation machinery to skip over exon 13 in the *USH2A* gene. This trial has progressed to phase 1/2 with evidence of effectiveness (NCT 03780257). However, the ProQR treatment has limited applicability, only targeting mutations within exon 13. Additionally, skipping certain exons results in a deleterious frameshift, and the treatment effect is temporary. A widely applicable, safe, and effective treatment does not currently exist for patients with Usher Syndrome.

The cDNA sequence of the *USH2A* gene (15.7 kb) exceeds the 4.7 kb packaging limit of AAV, eliminating the possibility of previously characterised gene augmentation approaches. Consequently, CRISPR (clustered regularly interspaced short palindromic repeats) treatments that edit the mutation at the DNA or RNA level are being explored. Moreover, there are limitations to gene replacement therapies that encourage the exploration of alternative treatments. Exogenously expressed transgenes demonstrate a declining treatment effect, and theories for this include the silencing of the exogenous transgene and cellular stress from products of the mutant allele [9,10].

This review evaluates the three CRISPR technologies in most frequent use: CRISPR/Cas9, base editing, and prime editing, for their potential to secure research funding for Usher Syndrome. The review will provide a summary of each tool, then proceed to explore their therapeutic potential in terms of applicability to combat thousands of described pathogenic *USH2A* mutations, safety, efficiency, and ease of in vivo delivery.

## 2. Tools: Summary of the Three CRISPR Technologies in Most Frequent Use

CRISPR represents a form of prokaryotic adaptive immunity employed by bacteria and archaea, which has been repurposed for use in gene therapeutics. Prokaryotes store short DNA sequences derived from invading bacteriophages. A variety of species-specific Cas proteins use the short DNA sequences as a guide to bind and cleave complementary bacteriophage DNA in the event of repeat invasions. The CRISPR/Cas9 system is the original prototype that has undergone successive modifications to generate other popular gene therapy tools such as CRISPR interference/activation, base editors, and prime editors. All three CRISPR technologies have strong potential to treat patients with Usher Syndrome.

### 2.1. CRISPR/Cas9: Summary

**Figure 2:**
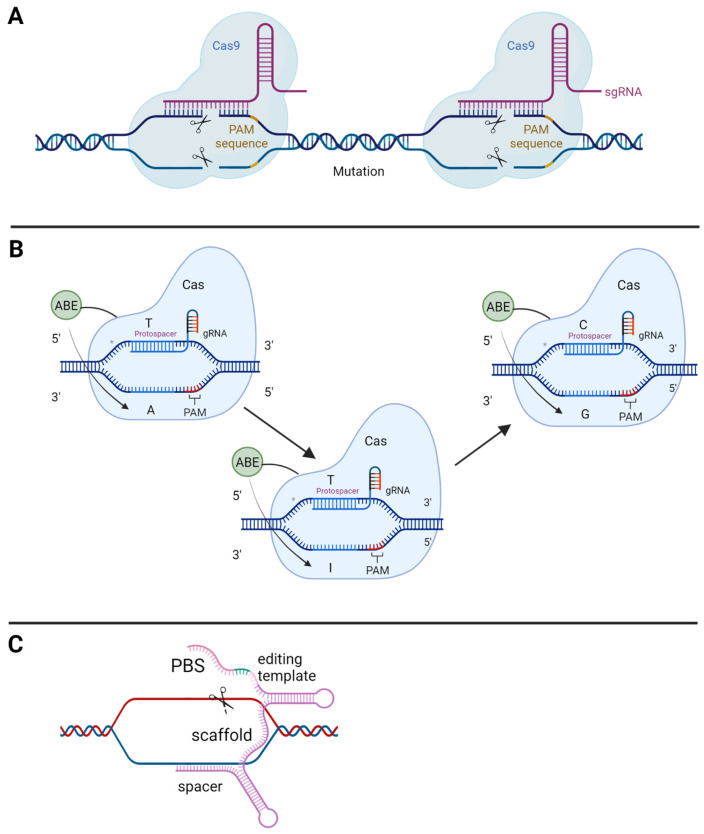
CRISPR Tools (**A**) CRISPR/Cas9: The CRISPR/Cas9 system mediates double-stranded breaks at the target site. Repair via the non-homologous end-joining mechanism leads to unpredictable insertion deletions. (**B**) Base editing: The base editing system directs an enzyme to the target region, where it mediates nucleotide transitions. Adenine base editors instigate a transition from adenine to inosine, which is recognised by the cell as guanosine. (**C**) Prime editing: The Prime editing system uses a Cas nickase to nick the target strand. * PBS (primer binding site). Created with Bio-Render.com (accessed on 10 January 2023).

In the basic CRISPR/Cas model, a sgRNA guides a Cas nuclease to the target DNA, where it initiates a double-strand break (Figure 2A). The sgRNA has two component parts: tracrRNA, which acts as a scaffold for the binding of the Cas nuclease, and crRNA, which encompasses a 17–20 nucleotide sequence complementary to the target region. SpCas9 is derived from *Streptococcus pyogenes* and induces a double-stranded break 3 base pairs 5′ of an NGG PAM recognition site. Two pathways mediate repair: non-homologous end joining (NHEJ) and homology-directed repair (HDR). NHEJ ligates the broken ends in a template-independent manner, resulting in unpredictable indel events. HDR mediates much more precise correction, utilising single-stranded oligodeoxynucleotides (ssODN) for templated repair.

### 2.2. Base Editing: Summary

CRISPR/Cas9 has been adapted to generate the base editing system by catalytically inactivating the Cas nuclease and fusing it with a deaminase enzyme that mediates nucleotide transitions (Figure 2B). Critically, base editing avoids double-stranded breaks. Canonical cytosine base editors (CBEs) utilise a naturally occurring cytosine deaminase enzyme (APOBEC1) fused to dCas9. Following complementary binding of the gRNA to target DNA, local denaturation exposes an R loop in the non-complementary strand for deamination [11]. The result is a C to T edit via a uracil intermediate [12]. Unlike CBEs, a naturally occurring adenine base editing enzyme (ABE) acting on ssDNA was not readily available, however, researchers were able to evolve the enzyme via directed evolution (stimulated by a chloramphenicol challenge) of E.coli TadA (a tRNA deaminase acting on the single-stranded anticodon loop of tRNA to convert adenine to inosine) [13,14]. ABEs are capable of mediating A to G transitions via an inosine intermediate. Newer generations of base editors have dual adenine and cytosine base conversion abilities [15] or C to G transversion capabilities [16].

### 2.3. Prime Editing: Summary

The use of a Cas nickase, which only cuts one strand of a double-stranded DNA template, and further modifications to the sgRNA allow the reconstruction of a DNA sequence through a repair mechanism on the cut strand in a process known as prime editing (Figure 2C). A reverse transcriptase (RT) template and primer binding site are added to the sgRNA to generate the pegRNA. The spacer region and primer binding region hybridise into opposite DNA strands, improving the binding specificity of the construct and reducing off-target edits. The Cas9 nickase cuts one strand of the DNA 3bp 5′ of the PAM site, and the 3′ hydroxyl group primes extension of the 3′ flap by the RT enzyme, using the pegRNA as a template. The unedited 5′ flap is preferentially cleaved by FEN1 nuclease [17].

## 3. Applicability: Analysis of the Potential of Each Tool to Correct the Ten Most Prevalent USH2A Mutations

The review will now consider which tool has the greatest potential for treating the largest patient group impacted by mutations within the *USH2A* gene (Table 1). In vitro experiments in relevant cells would be required to justify the use of these technologies at the gene therapy level.

### 3.1. CRISPR/Cas9: Applicability

*Targetable Mutations*

The CRISPR/Cas9 system is valuable for its ability to mediate large deletions and insertions. Precise sequence correction is possible via the HDR repair pathway but untenable when replying to NHEJ in non-dividing cells.

The ProQR commercial project has demonstrated that usherin retains its function after the antisense oligonucleotide (ASO) mediated skipping of exon 13 [18]. This is likely attributable to the repetitive structure of the protein in this region. Consequently, the c.2299delG, c.2276G>T, and c.2802T>G mutations, all of which lie within exon 13, are targetable with dual CRISPR/Cas9 guides that excise the full-length exon without inducing a frameshift. Introducing double-stranded breaks within the intronic region reduces the potential for interrupting the coding sequence: the EDIT-101 trial, which uses dual Cas9 guides to excise an intronic mutation, has demonstrated sufficient therapeutic safety to progress through clinical trial to treat Leber Congenital Amaurosis [19]. CRISPR/Cas9 could be applied to the c.7595-2144A>G mutation in a similar manner to excise a deep intronic mutation that generates a 152bp insert containing a premature stop codon (PSC) at an exon junction.

*Limitations*

It is unclear whether the other mutations located within different exons would be treatable by the same method. Research would have to evaluate the functional implications of each exon knockout. Alternatively, the point mutations: c.11864G>A, c.1256G>T, c.11156G>A, c.9799T>C, and the duplication c.920_923dup could theoretically be excised by two sgRNA’s flanking the affected codon. However, NHEJ in nondividing cells is likely to introduce unpredictable indels at the excision sites and could induce a frameshift. HDR would be the only feasible option. Excision of the mutated splice acceptor site in the c.8559-2A>G mutation is not a viable strategy unless a cryptic splice site further downstream in the exon could be used instead, or the exon is not essential to protein function.

### 3.2. Base Editing: Applicability

*Targetable Mutations*

Base editing enables precise nucleotide transitions (purine to purine or pyrimidine to pyrimidine). Around 30% of human pathogenic mutations are correctable by cytosine and adenine base editors; this extends to 37.3% of pathogenic *USH2A* mutations [20,21]. CBEs, mediating C>T edits on the sense or antisense strand can correct three *USH2A* mutations: c.8559-2A>G, c.9799T>C, and c.7595-2144A>G. A CBE could also correct the c.2276G>T mutation via the conversion of TTC (F) to TTT (F). Similarly, ABEs can correct the c.11156G>A and c.11864G>A mutations via direct A>G transitions. The other *USH2A* mutations can feasibly be solved by the replacement of the mutated codon with a conservative amino acid change (as opposed to a direct correction) that should not theoretically affect protein folding and function. Conservative amino acid changes are possible for the c.1256G>T, c.2276G>T, and c.2802T>G mutations: an ABE editor can mediate a TTT (F) to CTT (L) conversion for the c.1256G>T mutation; a TGG (W) to TCG/TCC (S) conversion for the c.2802T>G mutation; and a TTC (F) to CTC (L) conversion for the c.2267G>T mutation.

*Limitations*

Nonetheless, it is important to note that this analysis does not take bystander effects into consideration. Each base editing enzyme has a unique editing window when tethered to a unique species of Cas protein. This window will initially need to be validated to assess whether a suitable PAM site exists that positions the target base within the editing window without generating undesirable bystander events. Base editing has greater flexibility in the context of RNA editing, which does not require a PAM site. A notable limitation of base editing in contrast to CRISPR/Cas9 is the inability to mediate insertions or deletions. Consequently, base editors have no capability against the remaining two *USH2A* mutations: c.920-923dup or c.2299del.

### 3.3. Prime Editing: Applicability

*Targetable Mutations*

Prime editing offers a much broader applicability compared to CRISPR/Cas9 and base editing since it can mediate all base transitions in addition to small deletions of up to 80 base pairs and insertions of up to 44 base pairs [17,22]. Moreover, larger sequence alterations could potentially be introduced using multiple prime editors. The prime editing construct is less constricted by the PAM site: there is flexibility in the length of the RT template component of the pegRNA, considerably broadening its applicability relative to base editors. Prime editing tools enable the correction of every pathogenic point mutation within the *USH2A* gene: c.2276G>T, 2802T>G, c.11864G>A, c.1256G>T, c.11156G>A, c.7595-2144A>G, c.8559-A>G, and c.9799T>C. Additionally, targeted deletion within exon 6 enables correction of the c.920_923dup and targeted insertion can precisely correct the c.2299del.

Although prime editing has the greatest potential in terms of applicability for treating Usher Syndrome Type 2, it is vital to consider its practicality in a therapeutic setting. The review will now compare the tools in terms of efficiency, safety, and in vivo delivery.

## 4. Efficiency: Analysis of the Editing Efficiency Each Tool Achieves

It is important that the tool selected can accomplish levels of genomic editing sufficient for therapeutic rescue in vivo. The review will now discuss the potential of each tool in this respect.

### 4.1. CRISPR/Cas9: Efficiency

*Advantages*

CRISPR/Cas9 editing via the NHEJ pathway likely enables sufficient correction for phenotypic rescue in vivo, and several successful studies provide proof of principle evidence in favour of CRISPR/Cas9’s therapeutic potential. Large gene deletions in *DMD* restored dystrophin protein levels to ~8% of normal levels [23]. High levels of up to 50% editing have been detected in murine liver tissue, subsequently resulting in phenotypic changes such as the loss of F IX activity and emergence of a bleeding phenotype [24] or reduced blood cholesterol levels by 35–40% [25]. Additionally, a 27.9% correction has been achieved in an NHP model of LCA (Leber congenital amaurosis) [19]. The use of CRISPR/Cas9 exo-vivo has also generated promising results: knockdown of the BCL11A enhancer in autologous CD34+ stem cells eliminated vaso-occlusive episodes in TDT and SCD patients following bone marrow transplantation [26].

*Limitations*

Nonetheless, some studies have not achieved sufficient levels of correction via the NHEJ pathway for significant phenotypic results: 5% editing of stem cells in a patient with HIV and ALL was not enough to mitigate HIV infection [27]. The HDR pathway is likely too inefficient for precise correction of *USH2A* mutations in vivo as it targets nondividing cells such as photoreceptors very poorly [28]. Additionally, if precise correction is important, HDR has greater potential when delivered ex vivo into stem cells, an approach that is not yet validated in the retina. Efforts directed toward enhancing HDR editing efficiency have shown some success. Dual sgRNAs, the use of nocodazole to halt the cell cycle at G2/M, ssODN with asymmetric arms, and triple transfection protocols have improved editing rates when applied separately and simultaneously, achieving 38% editing rates of the *TNFa* gene [29,30]. It is possible that low levels of correction will alleviate signs of Usher syndrome: correction of the *Fah* gene in 1/250 murine liver cells was adequate for alleviating disease symptoms of hereditary tyrosinemia [30]. Nonetheless, a more efficient treatment would likely provide greater symptomatic relief.

### 4.2. Base Editing: Efficiency

*Advantages*

Base editing has also demonstrated proficiency for phenotypic rescue in vivo: constructs packaged within both single or dual vectors have shown levels of editing greater than 50% when delivered to animal models [31,32]. Moreover, editing levels achieved to date have translated to promising phenotypic improvement: in a mouse model of progeria syndrome, correction of a dominant negative C>T mutation more than doubled the lifespan of the mouse model [32]. The phenotypic improvement appears disproportionately high relative to levels of genomic editing: a 25% genomic correction in aortic tissue was correlated to an 11-fold increase in levels of vascular smooth muscle as well as reduced periadventitial fibrosis [32]. This may be partially attributable to an increase in editing rates with time [32].

The resounding evidence suggests that efficiency is not a limiting factor of base editing, and successive generations of base editing enzymes have successfully increased efficiencies further. Second-generation cytosine base editors (BE2) enhanced efficiencies threefold by tethering a uracil glycosylase inhibitor (UGI) to the construct to protect the uracil intermediate [12]. Inosine excision is inefficient, so a similar adjunct is not required for ABEs. Instead, phage-assisted continuous evolution (PACE) was applied to ABEs to increase their effectiveness, culminating in the ABE8e variant [33]. Third-generation base editors achieved a further two- to sixfold increase in efficiency by using an additional Cas9 nickase to nick the non-edited strand. The rationale is to guide the cell machinery to excise the unedited guanine and favour the uracil intermediate as a template for repair [12].

*Limitations*

Similar to CRISPR/Cas9 studies, editing rates show significant variability across different tissue types, ranging from 9-66% when targeting skeletal muscle compared to liver tissue [34,35]. Consequently, base editing may not be suitable for targeting certain tissues.

### 4.3. Prime Editing: Efficiency

*Limitations*

Low efficiency is a more significant limitation of prime editing compared to other CRISPR tools. Similar to base editing and CRISPR/Cas9, editing rates are highly variable across different genomic loci and cell lines (ranging from 0.3 to 80%) [36]. In vivo editing levels have not reached thresholds as high as other tools: prime editors have consistently shown genomic correction levels below 10%, with 6.4% in RPE tissue derived from a model of LCA and 1.71% correction of *Dnmt1* [37].

*Advantages/Solutions*

Nonetheless, good levels of in vitro editing exceeding 50% have been demonstrated, and some studies have achieved more promising in vivo efficiencies: 11.1% correction in a *Pah* model of phenylketonuria [36,38], 11.5% correction in liver tissue derived from a mouse model of hereditary tyrosinaemia [39], and 6.4–15.8% correction in hepatocytes from a human alpha-1 antitrypsin (AATD) model [40]. Moreover, when studies attempted a direct comparison across CRISPR technologies, base editing and CRISPR/Cas9 showed comparable efficiencies to prime editing at the same target location. An ABE demonstrated marginally higher levels of efficiency in RPE (11%) but lower levels in liver tissue (9.5%) [39]. The HDR pathway was found to be particularly inefficient in the LCA model (1.2%) and slightly less efficient in the hereditary tyrosinaemia model (9.3%) [39]. An additional study focused on delivering prime editors to liver tissue found levels of a correction lower than 10% (6.7% at 45 days post-injection) [40]. Even so, the levels of correction achieved with prime editors in vivo were sufficient for significant phenotypic changes to be observed.

Furthermore, prime editing is a more recent development than base editing, and efforts are ongoing to fully understand its mechanism and enhance efficiency. Second-generation prime editors increased editing efficiencies via an engineered reverse transcriptase, while PE3 variants nick the non-edited strand to increase efficiencies to 20–50% in HEK293T cells [17]. PE4 and PE5 variants transiently inhibit the mismatch repair pathway via a co-expressed the DNA mismatch repair inhibiting protein MLH1dn (MutL homolog 1) to increase levels of correction between 2 to 17-fold [41,42]. Additionally, modifying the pegRNA to increase exonuclease resistance and delivering sense and antisense pegRNAs that target the mutation from opposite strands have successfully enhanced editing efficiency [43,44]. It does appear that prime editing has the potential to instigate phenotypic rescue in vivo, and further efforts to enable the delivery of constructs that are not split across vectors could contribute to successful treatments.

## 5. Safety: Analysis of the Safety of Each Tool

Concerns are constantly raised over the safety implications of permanently editing patient DNA, particularly when considering vertical transmission (germline modification). Concerns typically relate to unwanted, often cumulative, off-target effects that could have serious unpredictable consequences, including tumorigenesis. It is important to weigh the cost/benefit related to a patient with a severely debilitating condition, and informed personal choice should play a role. It is also vital to select a tool that minimises off-target modifications with predictable action.

### 5.1. CRISPR/Cas9: Safety

*Limitations*

The CRISPR/Cas9 technology has significant safety implications that are poorly characterised. Indel events mediated by the NHEJ pathway may lead to undesirable frameshifts or frequent sequence changes within coding regions [45]. Three mismatches between the guide and DNA sequence are tolerated, increasing the likelihood of off-target events [46,47]. Moreover, double-strand breaks have been associated with large deletions, insertions, chromosomal truncations, and extensive rearrangements, termed ‘chromothripsis’ [48,49]. These events raise concerns over carcinogenicity, and double-stranded breaks have even been shown to directly activate the p53-mediated DNA damage pathway [50].

Several in vivo studies to date have reported substantial unintended genomic changes as a result of CRISPR/Cas9. In an in vivo model of ALS (Amyotrophic lateral sclerosis), CRISPR/Cas9 frequently induced large DNA deletions of hundreds to thousands of base pairs, mediated by proximally located identical sequences [51]. The rate of off-target effects was cumulative over time: 1.28% at 196 days compared to 2.27% at 585 days, highlighting the importance of strategies to silence activity once a therapeutic threshold has been reached. Other studies report low or no off-target events. However, these studies often have limitations. For example, one study used ultra-deep sequencing restricted to exons associated with cancer regulation and reported zero off-target events at 10 days following stem cell transfection [52]. However, a longer-term study is clearly required as well as an analysis of other exons/introns. Additionally, standard amplicon sequencing does not recognise more drastic events such as large deletions, insertions, or chromosomal translocations. It is important to assess the construct safety in vivo where an ex vivo approach is not intended (as in the retina).

*Solutions*

Solutions to the safety ramifications of CRISPR/Cas9 include self-inactivation of the vector as a function of editor accumulation (showing therapeutic effect) and using the Cas9 nickase variant to create a staggered double-strand break to improve specificity [53]. However, at present, the potential for catastrophic genomic changes and accumulation of unwanted off-target events over time pose a significant problem for CRISPR/Cas9-related Usher Syndrome therapies.

### 5.2. Base Editing: Safety

*Limitations*

Base editors have generally been promoted for their enhanced safety relative to CRISPR/Cas9, largely due to the avoidance of double-stranded breaks. However, off-target effects are a considerable limitation of base editing. The off-targets induced by base editors are classified as sgRNA-independent or sgRNA-dependent. Independent off-targets involve the interaction of the deaminase with genomic DNA: this rate can be concerningly high. The CBE BE3 was found to generate tens of thousands of off-target RNA editing events: rAPOBEC1 mutants with reduced binding ability and narrowed editing windows reduced but could not eliminate these events [54]. The ABE indel rate is significantly lower when compared to CBE’s [55], and this is proposed to result from inefficient deoxyinosine excision relative to deoxycytidine excision: spontaneous deamination of deoxyadenosine occurs at 2–3% the rate of deoxycytidine deamination, resulting in a slower selection pressure for the cellular enzyme mediating deoxyinosine repair [56]. Nonetheless, every embryo edited with an ABE7.10 variant still gained an average of 10 new SNVs [57].

Importantly, none of the sgRNA-independent off-target edits overlapped with software-predicted mutations, and SNVs were identified within both proto-oncogenes and tumour suppressor genes, highlighting an oncogenic risk. Notably, several papers have claimed to find no [58] or low (up to 0.45%) [35] off-targets in sites predicted by software such as Cas-OFFinder; however, these studies have neglected to examine sgRNA-independent effects. SgRNA-dependent off-target edits occur at predictable sites with sequence similarity to the target region and include bystander edits in proximity to the target nucleotide. It is predicted that 47% of protein-coding genes edited with canonical ABE and CBEs are susceptible to bystander edits: acquisition of nonsense or missense mutations could have catastrophic consequences.

*Advantages/Solutions*

Unlike CRISPR/Cas9 or prime editing, base editing has RNA editing capabilities. This has marked safety advantages, entailing a temporary effect without the risk of vertical transmission. One drawback is that the temporary nature of RNA editing would necessitate repeated therapeutic injections, and it is possible that patients may mount an immune response over time, neutralising the editing construct. Cas9-specific antibodies have been detected in adult mice following injection, suggesting the development of an adaptive immune response [38]. Some solutions to off-target accumulation include delivering the base editors or sgRNA in the form of mRNA [59] or RNP [33]: faster degradation times limit the enzyme’s genomic interaction. In addition, high-fidelity nCas9 variants with greater target specificity are being developed [60]. Nonetheless, current base editors have an off-target rate that is undesirable for therapeutic applications.

### 5.3. Prime Editing: Safety

*Advantages*

Prime editing has a lower rate of indel formation and unintended off-target edits compared to CRISPR/Cas9 or base editing technologies: it does not encounter the problem of bystander edits and is capable of highly precise sequence correction [17,22]. Some studies have reported a complete absence of off-target base substitutions using whole genome sequencing [61,62] or with Digenome-Seq and Cas-OFFinder software at predicted sites in both *rd12* and *Pah* mouse models [39]. Other studies have reported low rates of off-target editing (0.1–1.9% in 5 of 9 predicted sites in vitro) [63], 0.17% at *Dnmt1* in retinal cells [37], and 0–0.23% off-targets in 179 predicted sites in plant cells [62].

*Limitations*

The PE3 system, incorporating an additional nicking sgRNA, appears to result in higher rates of off-target editing: ~6.6% of modified alleles contained unintended indels in one in vivo study [40] and up to 3.8% in hepatocytes of a human AATD model [40]. In terms of fourth and fifth-generation prime editors, inhibition of mismatch repair (MMR) has implications for genomic mutations and tumorigenesis [64]. A long-term study of transient MMR inhibition mediated by PE4 and PE5 systems is required to fully assess the safety implications such that a compromise can be found between safety and efficacy. Nonetheless, the prime editing tools (especially the PE1 and PE2 systems) appear to edit in a more precise and targeted manner than other CRISPR technologies and offer better safety levels at present.

### 5.4. Comparative Summary

Prime editing appears to be the most promising CRISPR tool based on previous in vitro studies, showing the broadest applicability, highest safety levels, and comparable editing efficiencies. However, levels of in vivo editing are hampered by the large size of the construct and the inability to currently package prime editors within a single AAV vector. Consequently, this review will now evaluate alternative delivery options and the implications for therapeutic success.

## 6. Delivery Mechanisms: Analysis of Potential Delivery Systems to Deliver CRISPR Tools

### 6.1. Viral Delivery

*Advantages*

The AAV vector arguably represents the most effective CRISPR delivery tool from a safety and efficacy standpoint. In more than one hundred clinical trials that used the AAV vector, there was no evidence of genotoxicity [65]. The FDA deemed AAV adequately safe to approve its use in delivering gene therapy to LCA patients. The cell specificity is a particular advantage: capsid tropism constricts activity to certain tissue types, and this can be further enhanced by introducing miRNA target sites into the 3′UTR, enabling endogenous microRNAs to act as transcriptional repressors where activity is undesirable [29,66].

*Limitations*

However, there are notable drawbacks associated with AAV delivery. Integration within host DNA is technically possible, even with recombinant (rAAV) vectors that lack the *Rep* gene, due to random recombination events [67]. In mouse models predisposed to hepatocellular carcinoma, rAAV integration has been linked to enhanced oncogenesis [68]. A small number of patients with pre-existing liver disease enrolled in a clinical trial have died as a direct result of receiving super-high doses of AAV that induced liver dysfunction and sepsis [69]. Doses used for retinal transduction are many log units lower, although the potential for a systemic effect should be investigated and the ‘immune privileged’ status of the eye better characterised.

Moreover, AAV is not applicable to all patients due to pre-existing neutralising antibodies against certain AAV serotypes, limiting the treatment effect [70]. Additionally, patients receiving RNA editing tools that require frequent injection may mount an immune response over time. It could be possible to circumvent this to a degree using an algorithm termed ‘SCHEMA’: the algorithm generates new capsid variants with greater tissue specificity, reduced susceptibility to neutralising antibodies, and greater transduction efficiency [71].

When considering the applicability of CRISPR/Cas9 delivery into the retina, AAV2/8 serotypes, in particular, show good levels of photoreceptor transduction [72]. The difficulty lies with the strict 4.7kb packaging limit of the viral vector. CRISPR/Cas9 tools can be comfortably packaged, however, the size limit is particularly problematic for the delivery of the more recent CRISPR tools with larger transgene sequences. Solutions to this include tethering enzymes to smaller Cas variants, however, these typically impact the efficiency [40]. Alternatively, dual vector approaches have been trialled. Base editing constructs packaged within dual AAV vectors can mediate phenotypic rescue in vivo [32]. Dual vector packaging of prime editors is possible with truncated reverse transcriptase variants, however, split plasmid approaches lower activity [35,40,73].

### 6.2. Electroporation

*Advantages*

The broad applicability of prime editing for the correction of *USH2A* mutations alongside the packaging limitations of AAV encourages the exploration of alternative delivery approaches. Retinal electroporation involves passing an electrical current across the retina using subretinal tweezer electrodes. The negatively charged DNA travels into the retina and enters the photoreceptors through temporary cell membrane pores. Electroporation has shown in vivo efficacy to date: rodents receiving CRISPR/Cas9 therapy via retinal electroporation displayed a successfully edited rhodopsin gene in a model of autosomal dominant retinitis pigmentosa [74]. Additionally, electroporation of newborn mice with plasmids containing a GFP transgene generated ~80% GFP-positive rod photoreceptor cells, and the animals showed high survival rates despite their age and susceptibility to anaesthesia [75].

Therapeutic electroporation in vivo would rely on minicircle DNA (plasmid DNA with the prokaryotic elements removed from the backbone) to avoid transgene silencing [76]. Minicircle delivery via electroporation alleviates the tight size restrictions imposed by AAV packaging and holds significant promise for the delivery of larger prime and base editing constructs in addition to much larger cDNA sequences such as *USH2A*. The limitations of exogenous transgene expression are a consideration, although this does avoid permanent DNA editing.

*Limitations*

However, electroporation is likely to require extensive optimisation if intended for in vivo use in the human retina, especially since the high voltage shock required to permeabilise cell membranes can be toxic [77]. It is currently more suitable for ex vivo editing. Electroporation has shown greater efficiency for ex vivo editing than techniques such as pronuclear microinjection [78], and significant editing has been demonstrated in mouse zygotes [79]. There is potential to edit autologous embryonic or induced pluripotent stem cells via electroporation or otherwise, then differentiate them into retinal sheets for transplantation into the retina [80]. Beam Therapeutics has delivered base editors to autologous haematopoietic stem cells for reactivation of fetal haemoglobin in sickle cell anaemia and beta-thalassemia, and the FDA has approved progression to phase 2 clinical trial [81]. Additionally, 80% conversion of HBBs to HBBg in cells from SCD patients was achieved using ABE-NRCH [82]. This technique circumvents the packaging requirements of AAV and possibly limits the off-target or systemic impacts of CRISPR. Nonetheless, although grafts have demonstrated the ability to form synaptic connections with recipient cells in primate models of retinal degeneration, this approach is far from well-established, and the tumorigenic potential of stem cells is a considerable ramification [83,84]. In vivo retinal electroporation is a potential strategy; however, it is still in the early stages of research when compared to viral delivery, which has already entered the therapeutic domain.

### 6.3. Nanoparticle Delivery

*Advantages*

Nanoparticles have some distinct advantages compared to viral delivery: they are more economical, enable high loading levels, do not trigger an immune response, and there are no concerns over mutagenesis. They may be useful in patients with pre-existing neutralising antibodies to AAV or in patients receiving RNA editing treatments where it would be useful to avoid a mounting immune response with repeated injections. Successful in vivo editing has been demonstrated with this approach: RNPs encased within lipid nanoparticles restored dystrophin expression in DMD mice and significantly reduced serum PCSK9 levels in C57BL/6 mice [85]. As in electroporation, nanoparticle delivery holds promise for packaging prime and base editing constructs or larger cDNA sequences. DNA nanoparticles containing the RPE65 transgene and S/MARs were capable of transducing retinal pigment epithelium to mediate structural and functional improvements in an LCA mouse model [86].

*Limitations*

However, lipid nanoparticles generally demonstrate low editing efficiencies, and the packaging of CRISPR/Cas9 plasmids within LNPs has not met clinical thresholds [87]. Gold nanoparticles encasing Cas9 RNP have demonstrated improved editing levels of ~30% in vitro. Nonetheless, toxicity is a concern: gold nanoparticles, in particular, result in toxicity at high concentrations where they have a tendency to form aggregates [88]. Moreover, nanoparticles have not proven capable of photoreceptor transduction to date and are not currently an option for *USH2A* editing in the retina.

### 6.4. Comparative Summary of Different Delivery Mechanisms

AAV delivery is currently the best option for the delivery of gene therapy into the retina: the safety has been extensively validated through clinical trials, and transduction is organ-specific. However, it has notable drawbacks, specifically the potential for oncogenesis and immunogenicity in addition to strict packaging requirements. Prime editing arguably has the greatest potential for the treatment of *USH2A* mutations. However, prime editors cannot currently be packaged within a single AAV. Further research is required to optimise and authenticate in vivo delivery of prime editors via electroporation or nanoparticles. The output could be very widely applicable for improving human health in conditions that have a strong genetic basis. The efficiency, safety and in vivo delivery potential for each CRISPR tool are compared in Table 2. 

## 7. Conclusions

Many genetic diseases, including Usher Syndrome, are characterised by a markedly heterogeneous database of mutations. The result is that clinical trials will struggle to acquire funding for treatments with a narrow reach directed toward rarer variants. An approach to maximise funding would be to focus on treatments with broad applicability that can be easily adapted to different mutations.

Prime editing has the broadest applicability of all current CRISPR technologies and represents a promising focus for future research efforts. It can correct all ten of the most common mutations in the *USH2A* gene due to its ability to mediate all base transitions as well as small deletions and insertions. In contrast, the CRISPR/Cas9 system can only definitively correct four of the ten most common *USH2A* mutations via intronic deletions and has the potential to correct five more depending on the importance of the involved exon to protein function. The precise correction is possible with prime editing but is unlikely to be a viable option with CRISPR/Cas9 strategies since HDR is very inefficient in non-dividing cells such as photoreceptors. Base editing can correct eight of the ten most common *USH2A* mutations, although some of these edits would mediate a conservative amino acid change rather than an exact reversion to the original codon. The remaining two mutations are not targetable with base editors since they are unable to correct deletions or insertions.

In terms of therapeutic potential, safety and efficiency are also important considerations. Prime editing has superior safety and precision of editing relative to other tools. CRISPR/Cas9 mediates double-stranded breaks that can lead to indel events and catastrophic chromothripsis. The NHEJ process favoured in non-dividing cells repairs the broken DNA ends in an unpredictable manner. HDR has a much more precise mode of action but is generally only effective ex vivo. HDR of autologous stem cells and subsequent differentiation into retinal progenitor cells is a possibility, however, the tumorigenic potential is a real concern, and retinal stem cell therapy has not proven to be a viable strategy to date. The safety of base editing is also dubious: new SNVs are frequently introduced, and bystander editing entails unpredictable genomic changes that limit the precision of this technique.

Despite the clear advantages of prime editing from the perspectives of applicability and safety, the tool is hampered by its efficiency and delivery in vivo. CRISPR/Cas9 and base editors are both packageable within a single AAV vector, whereas prime editors are only packageable within dual vectors, which considerably lowers activity due to the recombination step following transfection. AAV is a relatively safe, FDA-approved vector that is readily available. Other delivery tools, such as electroporation, still require extensive optimisation for use in human patients, prompting the reconsideration of other CRISPR tools in favour of prime editing. Nonetheless, further research would still be required to improve the safety of CRISPR/Cas9 and base editing tools.

Research efforts would perhaps be better directed towards creating more compact prime editing constructs or developing more robust delivery methods with greater capacity, with a focus on maximising the size of the treatment population. This would ultimately generate tools that are highly attractive to investors and maximise funding for research into rare diseases.

## Figures and Tables

**Table 1:** Table to show the ten most common pathogenic mutations causing Usher Syndrome Type 2A. Editing options are based on in silico predictions. Mutations recorded in the LOVD Database accessed 11 April 2023 (✓ possible, x not possible, ? uncertain possibility).

USH2A Mutation	Exon/Intron		Description	Treatment Options
c.2299del	13	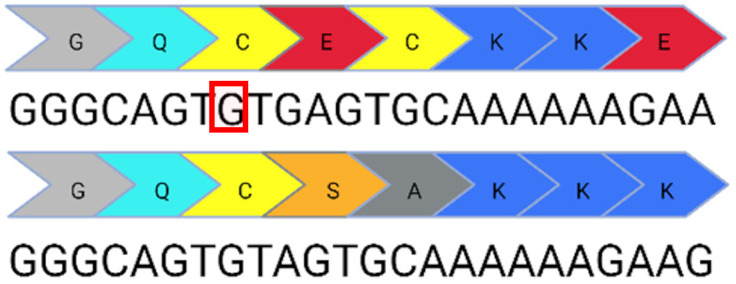	Deletion in G nucleotide	✓CRISPR/Cas9 x Base editing ✓Prime editing
c.2276G>T	13	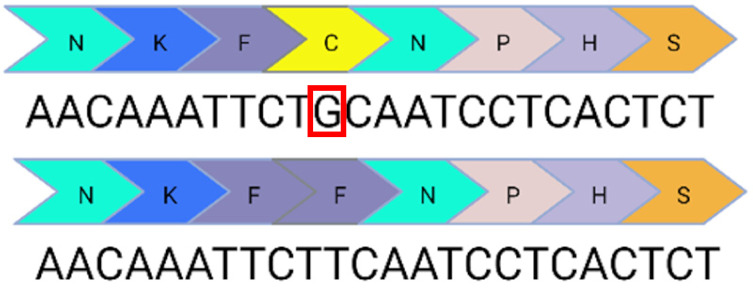	G to T point mutation causing Cys (C) to Phe (F)	✓CRISPR/Cas9 ✓CBE ✓Prime editing
c.2802T>G	13	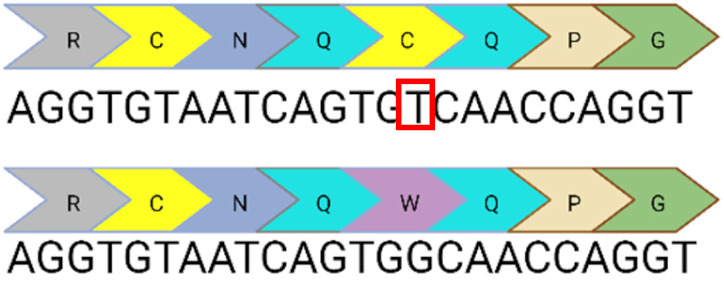	T to G point mutation causing Cys(C) to Trp (W)	✓CRISPR/Cas9 ✓ABE (conservative change) ✓Prime editing
c.11864G>A	61	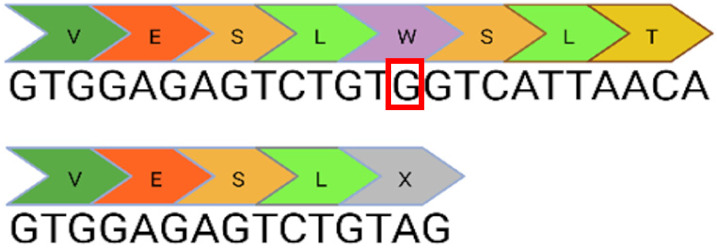	G to A point mutation causing Trp (W) to Stop (X)	? CRISPR/Cas9 ✓ABE ✓Prime editing
c.8559-2A>G	41i, 42i, 55	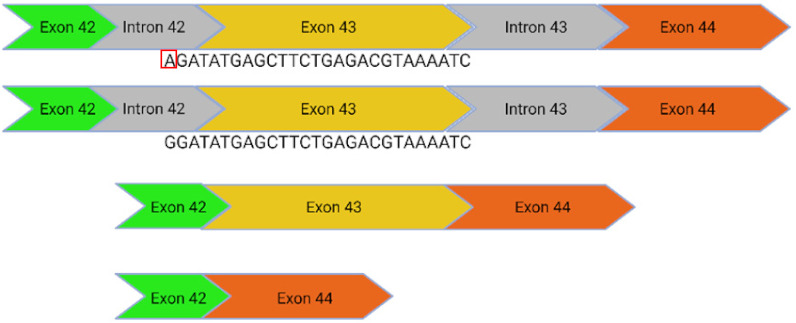	Affects acceptor splice site in intron 42 causing exon 43 skipping and 41 amino acid deletion	x CRISPR/Cas9 ✓CBE ✓Prime editing
c.7595-2144A>G	39i, 40i	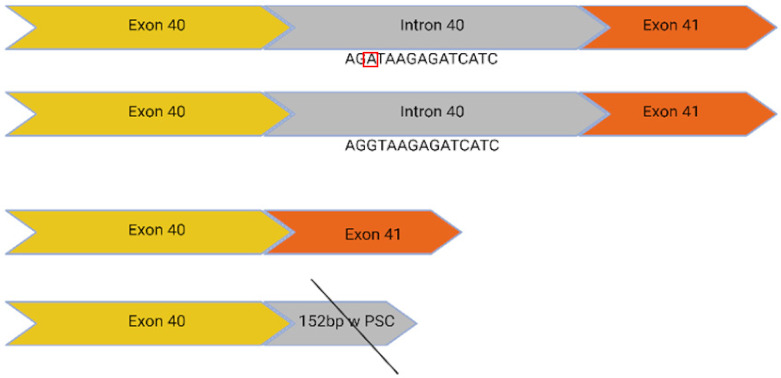	Deep intronic mutation generates a 152 bp insert containing a premature stop codon at the junction of exon 40 and 41	✓CRISPR/Cas9 ✓CBE ✓Prime editing
c.920_923dup	6	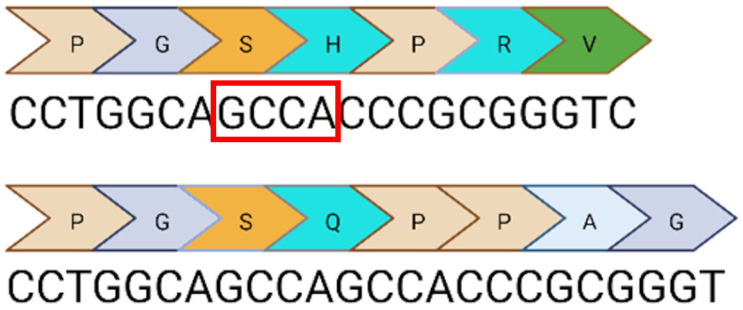	4 bp duplication introduces a 16 bp premature stop codon downstream	? CRISPR/Cas9 x Base editing ✓Prime editing
c.1256G>T	7	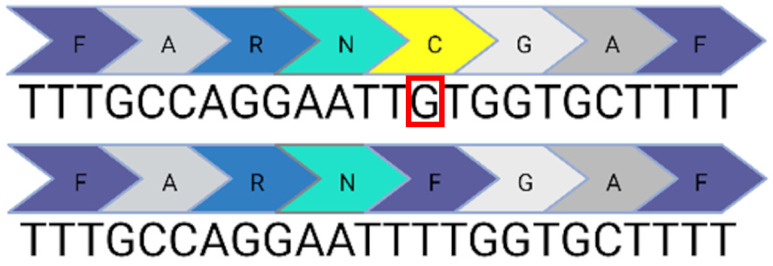	G to T point mutation causing Cys (C) to Phe (F)	? CRISPR/Cas9 ✓ABE (conservative change) ✓Prime editing
c.11156G>A	57	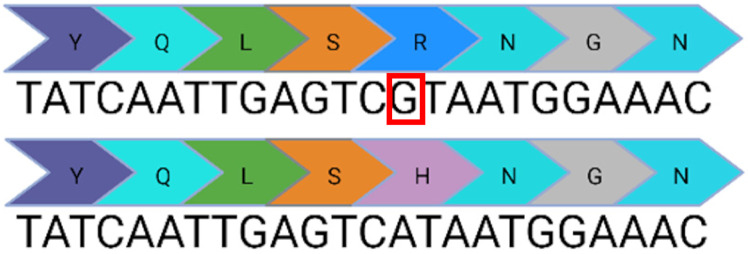	G to A point mutation causing Arg (R) to His (H)	? CRISPR/Cas9 ✓ABE ✓Prime editing
c.9799T>C	50	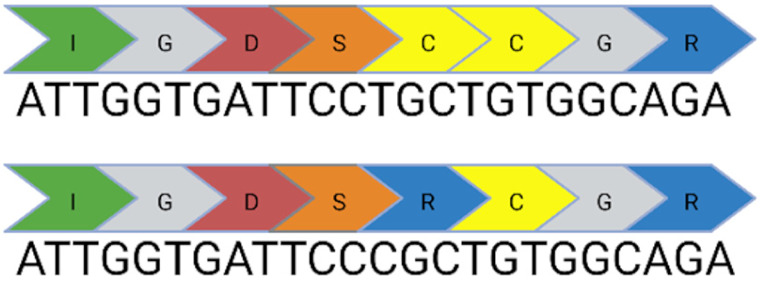	T to C point mutation causing Cys (C) to Arg (R)	? CRISPR/Cas9 ✓CBE ✓Prime editing

**Table 2:** Summary table comparing the efficiency, safety, and in vivo delivery options for CRISPR tools (✓ possible, x not possible).

	Efficiency	Safety	In Vivo Delivery
CRISPR/Cas9	▪ Up to 50% in vivo with NHEJ pathway ▪ Lower efficiency with HDR pathway ▪ Variable across tissue types	▪ Double strand breaks associated with large insertions, deletions, and chromothripsis ▪ Off-target effects up to 2.27% reported in vivo	✓ Viral x Electroporation: possible with an ex vivo approach, but not currently possible in vivo delivery into retina x Nanoparticles do not currently transduce photoreceptors
Base editing	▪ Greater than 50% editing demonstrated in vivo ▪ Variable across tissue types	▪ No double strand breaks ▪ 47% genes susceptible to bystander edits ▪ Some papers demonstrate low off-targets (<0.45%)	✓ Viral x Electroporation: possible with an ex vivo approach, but not currently possible in vivo delivery into retina x Nanoparticles do not currently transduce photoreceptors x Nanoparticles do not currently transduce photoreceptors
Prime editing	▪ Greater than 50% editing demonstrated in vitro ▪ In vivo editing generally lower than 20% ▪ Variable across tissue types	▪ No double-strand breaks ▪ No bystander edits ▪ Generally low off-target edits (0.1–1.9% reported)	✓ Possible with dual AAV vectors x Electroporation: possible with an ex vivo approach, but not currently possible in vivo delivery into retina x Nanoparticles do not currently transduce photoreceptors

## Abbreviations

Abbreviation	Meaning
AATD	alpha-1 antitrypsin
AAV	adeno-associated virus
ABE	Adenine base editor
ALS	Amyotrophic lateral sclerosis
ASO	antisense oligonucleotide
CBE	cytosine base editors
CRISPR	clustered regularly interspaced short palindromic repeats
HDR	Homology directed repair
LCA	Leber congenital amaurosis
MMR	Mismatch repair
NHEJ	Nonhomologous end joining
NHP	Non-human primate
PACE	phage-assisted continuous evolution
PSC	Premature stop codon
RP	Retinitis pigmentosa
RPE	Retinal pigment epithelium
RT	Reverse transcriptase
ssODN	single-stranded oligodeoxynucleotides
UGI	uracil glycosylase inhibitor
rAAV	recombinant adeno-associated virus
